# The necessity of multi-parameter normalization in cyanobacterial research: A case study of the PsbU in *Synechocystis* sp. PCC 6803 using CRISPRi

**DOI:** 10.1016/j.jbc.2025.110763

**Published:** 2025-09-24

**Authors:** Maria Christine Veit, Ron Stauder, Yu Bai, Ragini Gabhrani, Matthias Schmidt, Stephan Klähn, Bin Lai

**Affiliations:** 1 BMBF Junior Research Group Biophotovoltaics, Department of Microbial Biotechnology, Helmholtz Centre for Environmental Research – UFZ, Leipzig, Germany; 2 Molecular Biology of Cyanobacteria Group, Department of Solar Materials Biotechnology, Helmholtz Centre for Environmental Research – UFZ, Leipzig, Germany; 3 Centre for Chemical Microscopy (ProVIS), Department of Technical Biogeochemistry, Helmholtz Centre for Environmental Research – UFZ, Leipzig, Germany

**Keywords:** chlorophyll, CRISPR, cyanobacteria, photosynthesis, photosystem II, polymerase chain reaction, gene interference, cell diameter, cell morphology

## Abstract

Photosystem II (PSII) is a multiprotein complex and plays a central role in oxygenic photosynthesis. PsbU, a 12 kDa subunit of PSII, is associated with thermotolerance and structural stabilization of the oxygen-evolving complex in cyanobacteria. Corresponding knockout strains showed decreased oxygen evolution rates, although the growth was not impaired. In this study, we provide further insights into the consequences of PsbU perturbations and propose to revisit the impact of PsbU on cell physiology. We made use of CRISPRi to knock down the *psbU* gene in *Synechocystis* sp. PCC 6803, and assessed previously described effects referred to different biomass parameters including optical density, chlorophyll a content and cell number. After knocking down *psbU,* the growth rate was decreased by 15% based on counting the cell number, while this effect was not observed when monitoring optical density. Furthermore, the oxygen evolution rate per cell in the *psbU* knockdown strain did not show a significant difference compared to the control groups, which was probably due to its larger cell size and higher chlorophyll *a* content per cell. The decreased quantum efficiency of pigments was compensated by the increased pigment content on the single-cell level in the knockdown strain. Our results complement previous analyses and highlight the importance of evaluating cyanobacterial physiology based on different biomass quantitative units to avoid misinterpretation of the results.

Oxygenic photosynthesis plays a crucial role in contemporary biogeochemical cycles (1). This process combines two multiprotein complexes, photosystem I (PSI) and photosystem II (PSII), that enable the conversion of light into chemical energy and the generation of a sufficient redox potential to oxidize water and utilize it as electron source (2). Light-driven water oxidation releases oxygen as a byproduct, which is the basis for aerobic life. The obtained electrons are used to fix and reduce CO_2_ to generate organic carbon compounds. Oxygenic photosynthesis is the major pathway for primary production (3) and hence, the fundament of feeding heterotrophic organisms such as animals and subsequently humanity on earth. Apart from their ecological role, oxygenic photoautotrophic organisms (*e.g.* cyanobacteria, algae, and plants) are also utilized in pharmaceutical and industrial biotechnology. In the past decade, tremendous efforts have been put into advancing the field of photobiotechnology using phototrophic microorganisms for various applications including *e.g.* the production of food, chemicals or energy carriers (4, 5, 6, 7).

Central to oxygenic photosynthesis is PSII, which catalyzes the solar-driven water-splitting reactions. PSII is a large protein complex, consisting of over 20 subunits (8). Intensive research has been done to understand the function of PSII and its subunits conserved in cyanobacteria and in chloroplasts of algae and plants in the past decades (9). The two core subunits D1 and D2 form a heterodimer that binds all key redox cofactors involved in internal electron transfer (10). Light energy is absorbed and directed toward the reaction center chlorophyll P680 by the chlorophyll-binding antenna proteins CP43 and CP47 (11). Upon light excitation, electrons are transferred to the nearby pheophytin (12) and subsequently enter the electron transport chain through the plastoquinones Q_A_ and Q_B_ (13). Finally, the electron deficit at P680 is replenished by the oxygen-evolving complex (OEC), which contains a Mn_4_CaO_5_ cluster.

Naturally, the alteration or disruption of such key components causes clear and profound changes in photosynthetic activity including decrease or loss of photoautotrophic growth and/or photosynthetic oxygen evolution. Such were extensively observed for mutants of the cyanobacterium *Synechocystis* sp. PCC 6803 (hereafter *Synechocystis*) lacking CP43, CP47, or D2 (14). Intriguingly, deletion of less conserved subunits, mainly luminal extrinsic polypeptides responsible for the correct ion composition proximal to the OEC (15), often results in less drastic physiological changes, despite their known crucial role for a stable architecture of the OEC (16, 17).

The stability of the OEC was reported to correlate with the subunit PsbU (16). PsbU is a small protein of 12 kDa, and is widely conserved in cyanobacteria, but lost in higher plants (18). Its function was found to be correlated with the presence of Ca^2+^ and Cl^-^ ions. A *Synechocystis* mutant lacking the *psbU* gene grew similar to the wild type in standard BG11 medium (16, 17), while its growth was severely impaired if either or both of the two ions were absent from the medium (18, 19). Further studies found PsbU to play a regulatory role in maintaining the transition state of the Mn_4_CaO_5_ cluster in the OEC (20), whose function also requires chloride ions as a cofactor (21). Accordingly, a diminished photosynthetic activity and oxygen evolution capacity by 20 to 60% was reported after knocking out *psbU* (16, 17). Furthermore, the knockout of *psbU* in *Synechocystis* decreased the stability of the PSII, particularly for the mutant with a residue mutation of the chlorophyll *a* (chl *a)*-binding protein CP47 at position 363 (22). All these observations strongly suggested an effect of PsbU on the stability of the OEC and subsequently oxygen evolution.

In this study, we found it is of critical importance to assess the biochemical function of a protein in cyanobacteria using multi-parameter normalization approach. We used an inducible CRISPRi technique to knock down the *psbU* gene in *Synechocystis* (23), measured and normalized the physiological impacts of PsbU perturbations on cell physiology using a comprehensive approach, including culture optical density, Chl *a* content, cell size and number. While using a single guide RNA (sgRNA) targeting the *psbU* sense strand, CRISPRi led to almost complete inhibition of the *psbU* gene at transcription level. Similar to previous reports on *psbU* knockout strains (16, 17, 18, 19, 20, 24), our *psbU* knockdown strains also did not show altered growth, when considering the optical density of cultures. However, when tracing the cell number directly, a clear decrease in the growth rate was measured, while the cell size and the Chl *a* content per cell increased. More importantly, the oxygen evolution rate normalized to either cell optical density or Chl *a* content decreased, but no effect was observed if normalizing the rate to the cell number. These results provide new insights into the physiological role of PsbU on the oxygen evolution of photosynthetically active cells, while at the same time, suggest the importance of a comprehensive determination of biomass parameters from different aspects, which could lead to a distinct conclusion.

## Results and discussion

### CRISPRi enables an inducible knockdown of the *psbU* gene

**Figure 1:**
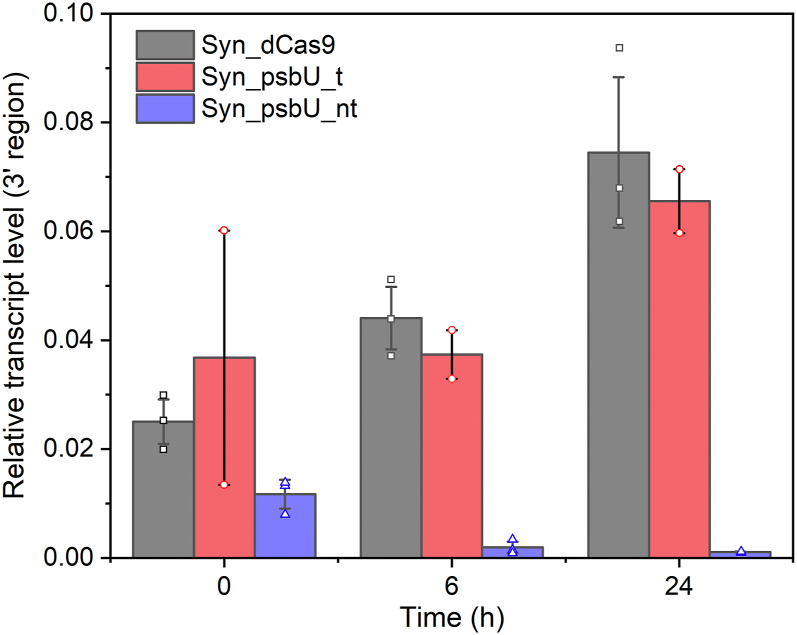
**Relative transcript levels of the 3′ region of *psbU* in different *Synechocystis* strains analyzed by RT-qPCR.***Syn*_dCas9: empty vector control; *Syn*_psbU_t: guide RNA targeting on antisense strand (also named template strand); *Syn_*psbU_nt: guide RNA targeting on sense strand (also named non-template strand). The mean and standard deviations were calculated based on: three biological replicates and three technical replicates for *Syn*_dCas9 and *Syn_*psbU_nt; two biological replicates and three technical replicates for *Syn*_psbU_t.

We designed two sgRNAs to target the dCas9 protein either to the sense (also named non-template strand) or antisense strand of the *psbU* gene locus, as in different case studies one or the other way was reported to be more effective in *Synechocystis* (23, 25, 26). The obtained *Synechocystis* strains, *Syn*_psbU_nt and *Syn*_psbU_t, were analyzed regarding the relative transcript levels of *psbU* after inducing the synthesis of both dCas9 and the sgRNA by anhydro-tetracycline (aTc) (Fig. 1). In the mutant strain with the sgRNA targeting *psbU* on the sense strand (*Syn*_psbU_nt), *psbU* transcript abundance was significantly decreased compared to the empty vector control strain harboring no *psbU*-specific sgRNA (*Syn*_dCas9). After 24 h, the *psbU* mRNA was barely detectable, indicating a strong knockdown of the *psbU* gene at the transcriptional level. Furthermore, it should be noted that the *psbU* transcript level was already lower prior to induction (time point 0 h), indicating that the CRISPRi system was active, even without the inducer, while the L_22_ promoter used in this system was reported to be tightly controlled by aTc (23, 27). In contrast, targeting the antisense strand of *psbU* did not show a significant effect, as the *psbU* transcript remained detectable in strain *Syn*_psbU_t strain at a similar level as in the control strain. Nevertheless, our results demonstrate that targeting the sense strand by CRISPRi is sufficient to constrain the transcription of *psbU,* which likely also affects *de novo* synthesis of PsbU protein, in turn resulting in physiological changes similar to previous reports.

### Knocking down *psbU* increases cell size and Chl *a* content per cell

**Figure 2:**
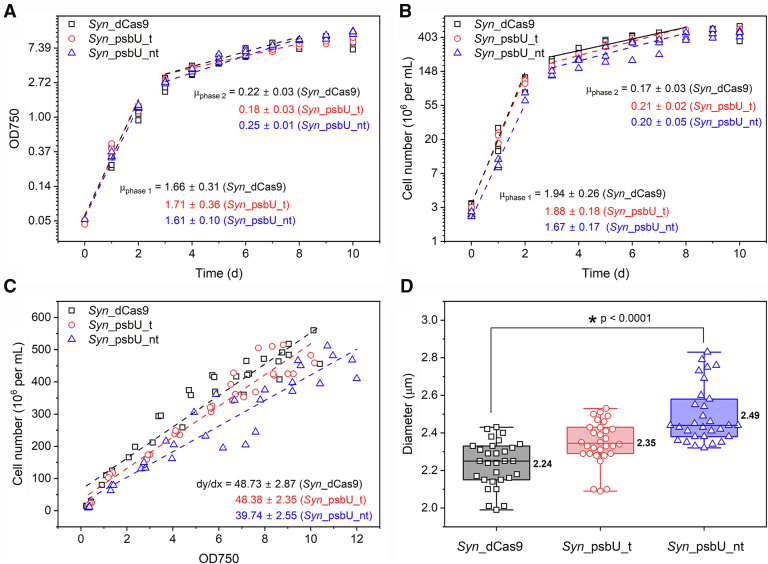
**Growth kinetics of different *Synechocystis* strains.***A*, growth rate based on the OD_750nm_ measurement. *B*, growth rate based on the cell number counting. *C*, correlation between OD_750nm_ and cell number. *D*, cell diameters of different *Synechocystis* strains over the whole cultivating batch. *Syn*_dCas9: empty vector control; *Syn*_psbU_t: guide RNA targeting on antisense strand; *Syn_*psbU_nt: guide RNA targeting on sense strand. Data was collected from three independent biological replicates. The statistics in panel *D* were done using Origin 2024b, two-sample *t* test.

The impact of the diminishing *psbU* transcription on cellular physiology was further assessed focusing on growth kinetics. When considering optical density of cultures, the tested strains *Syn*_dCas9, *Syn*_psbU_t and *Syn*_psbU_nt did not show a significant difference in growth rates (Fig. 2*A*), consistent with previous studies (16, 19). However, the cell morphology and size of cyanobacteria have previously been shown to be dynamic over different growth phases (28), posing challenges to determine the real growth status only *via* optical density measurement. By definition, microbial growth is defined as the proliferation of one cell into two daughter cells. Remarkably, a decrease in growth rate was observed for strain *Syn*_psbU_nt, when monitoring cell numbers (Fig. 2*B*). The maximum growth rate for the control strain was 1.94 ± 0.26 h^−1^ whereas strain *Syn*_psbU_nt showed a decreased growth rate of 1.67 ± 0.17 h^−1^ during exponential growth phase (phase I). These results demonstrate that PsbU-impaired *Synechocystis* strains show lower growth rates under standard conditions, which is visible only when considering cell numbers. This also demonstrates the necessity of multi-parameter analysis when investigating the growth of cyanobacteria.

The changes in cell number-based growth kinetics correlated with the cell morphology, as suggested by the analysis of cell number and diameter. The coefficient between cell number and optical density decreased by about 20% after *psbU* being knocked down, from 48.73 ± 2.87 ∗10^6^ cells/ml per OD750 for the control to 39.74 ± 2.55 ∗10^6^ cells/ml per OD750 for the *Syn*_psbU_nt strain (Fig. 2*C*). This result indicated that the cell volume increased upon *psbU* knockdown. This was indeed confirmed by analyzing the averaged cell diameter using a Coulter Counter (Fig. 2*D*). After knocking down *psbU*, the mean cell diameter increased from 2.24 μm to 2.49 μm (*p* < 0.0001). In addition, these data were supported using helium ion microscopy, which clearly conformed the increased cell size of strain *Syn*_psbU_nt (Fig. S2).

In addition to the cell size, the Chl *a* content was also affected by the *psbU* knockdown. The coefficient of Chl *a* content per unit optical density showed a general increasing trend when comparing the control strain with strain *Syn*_psbU_nt (see Fig. S1). While further being normalized to cell numbers, the Chl *a* content (μg per million cells) increased from 0.059 ± 0.005 to 0.080 ± 0.007 after knocking down the *psbU*. The lower abundance of PsbU protein led to about 35.6% increase in the Chl *a* content in single *Synechocystis* cells. The increase in Chl *a* content might lead to a change in the microbial membrane structures, contributing to the increase of surface roughness observed for the PsbU mutant *Syn*_psbU_nt (Fig. S2).

A summary of the growth kinetics and morphology parameters is given in Table 1.

**Table 1:** Growth kinetics and morphological parameters of *Synechocystis* strains

Strains	Growth rate μ [h^-1^]		Cell density per OD750 [10^6^ cells/ml]	Cell size [μm]	Chl *a* [pg/cell]
	OD750	Cell no.			
*Syn*_dCas9	1.66 ± 0.31	1.93 ± 0.26	48.73 ± 2.87	2.24	0.059 ± 0.005
*Syn*_psbU_t	1.71 ± 0.36	1.88 ± 0.18	48.38 ± 2.85	2.35	0.062 ± 0.005
*Syn*_psbU_nt	1.61 ± 0.10	1.67 ± 0.17	39.74 ± 2.55	2.49	0.080 ± 0.007

The changes of cell size and Chl *a* content caused by the *psbU* knockdown suggested that the PsbU subunit is not only contributing to the stability and thermo-resistance of the PSII activity, as previously reported (16, 24), but also is involved in the control of Chl *a* content and cell morphology, whereas the causal mechanisms remain to be revealed. Also, based on the cell number measurement, the knockdown of *psbU* in the PSII slowed down the growth rate already at 30 °C, even in the presence of Ca^2+^ and Cl^-^. This is likely due to the increase in cell size that requires more resources and, hence, has higher energy costs for biomass production.

### PsbU perturbation does not change the oxygen evolution rate per cell but lowers the quantum yield of chlorophyll

**Figure 3:**
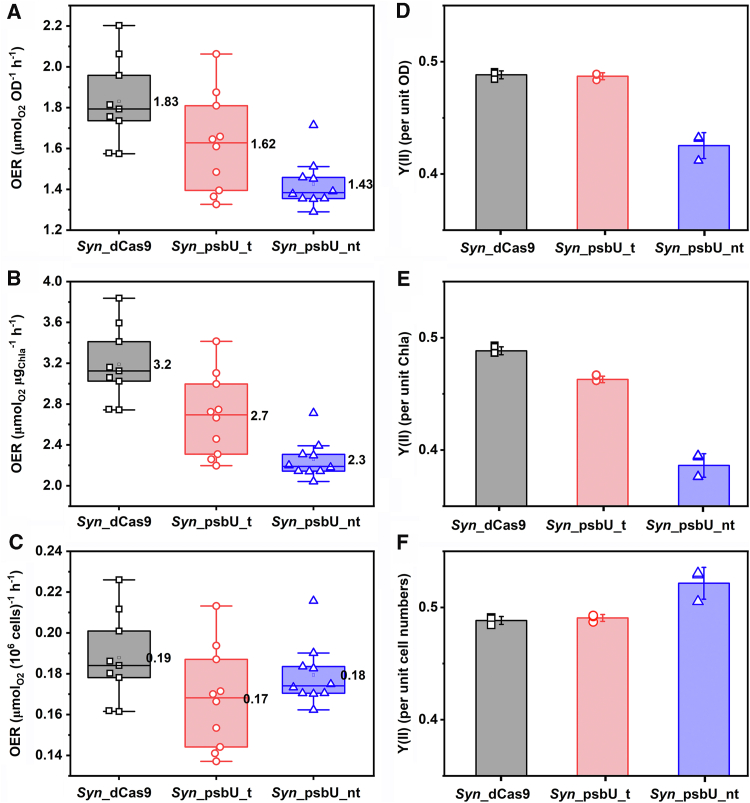
**Oxygen evolution rates (OER) and quantum yield Y(II) of different*****Synechocystis*****strains.** (*A* and *D*) OER and Y(II) normalized to OD_750_; (*B* and *E*) OER and Y(II) normalized to Chl *a* amount; (*C* and *D*) OER and Y(II) normalized to cell number. The OER measurements were performed 3 days after induction with 1 μg/ml aTc. The sample numbers for *Syn*_dCas9, *Syn_*psbU_t and *Syn*_psbU_nt were 9, 10 and 10 respectively. The quantum yield was measured at 50 μmol/m^2^/s 1 day after induction with 1 μg/ml aTc. The sample size was three independent biological replicates for all strains.

PsbU was reported to be essential for the functional stability of the OEC in PSII, and a lack of this subunit resulted in a decreased oxygen evolution rate per unit Chl *a* (16, 17). This conclusion is also supported by our work (Fig. 3, *A* and *B*). After normalization to the optical density or Chl *a* content, the oxygen evolution rate decreased by 20% to 30% in the *psbU* knockdown strain *Syn*_psbU_nt compared to the control strain.

However, no significant difference in the oxygen evolution rate was observed among the three tested strains when normalizing to cell number (Fig. 3*C*). It seemed that the observable decrease in the oxygen evolution rate upon *psbU* knocking down, normalized to the parameters Chl *a* content or optical density, was mainly due to a decrease in cell numbers under these scenarios (Table 1). These apparently contradicting results indicate that *Synechocystis* cells could possibly have an internal mechanism to maintain stable photosynthetic capacity on a single-cell level. The increased cell size is accompanied by the increased amount of Chl *a* content, which ultimately leads to the same level of photosynthetic electron flows to drive cell metabolism. Further investigation on the bioenergetics of photosystems will be needed to understand this phenomenon better.

The quantum yields of the three strains after induction were also analyzed and normalized to the OD, Chl *a* content and cell number, respectively (Fig. 3, *D*–*F*). The effective photochemical quantum yield Y(II) correlates to the quantity of open PSII reaction centres and their efficiency for photon capture (29). Thus, disruption of PSII and OEC architecture would negatively impact Y(II) values. The knockdown of *psbU* in the *Syn*_psbU_nt strain resulted in a lower quantum yield compared to the values of the other two strains, while normalizing the quantum yield to the cell optical density (Fig. 3*D*) and Chl *a* content (Fig. 3*E*). The decreased quantum yield per unit Chl *a* content agrees with previous reports on PsbU as an important player in maintaining OEC activity of PSII. This could be due to either instability of PSII structure or an increased number of closed reaction centres that are unavailable for photochemistry (24). Accordingly, the contribution of fewer open and optimally functioning reaction centres consequently leads to an impaired oxygen evolution as shown in Figure 3*B*. The lower quantum yield at the time of inoculation again indicates the expression system is leaky, as already shown in Figure 1.

In contrast, the *psbU* knockdown strain *Syn*_psbU_nt showed a higher quantum yield per cell, compared to the other strains (Fig. 3*F*). Such contradictory results were likely due to the compensation from increased Chl *a* content per cell. As shown above, the knockdown of *psbU* decreased the quantum yield of Chl *a* content by ∼20%, but increased the absolute quantity of Chl *a* per cell by ∼35.6%. This again suggests *Synechocystis* cells seem to have an internal mechanism to compensate for the PsbU function in PSII by changing the morphology of the cells.

## Conclusion

The biochemical function of the PsbU subunit of PSII has been studied for many years in cyanobacteria. PsbU has been reported to affect the structural stability and thermostability of the oxygen evolution complex, leading to decreased oxygen evolution rates in corresponding *psbU* mutants. However, our findings suggested that these conclusions were not comprehensive. We applied CRISPRi to knockdown *psbU* transcription and evaluated growth kinetics and photosynthetic activities of the recombinant strains based on three different biomass criteria, *i.e.* optical density, Chl *a* content and cell numbers. The cell behaviors, while normalizing to either the optical density or the Chl *a* content, agreed well with the literature reports. However, while looking into the single-cell level, in particular, cell size and number, the conclusions would be different. The cell size of the *psbU* knockdown mutant was increased compared to the control strains. In turn and contradictory to literature reports, the knockdown of *psbU* did not change the specific oxygen evolution rate per cell. Although the quantum efficiency of the pigments decreased after knocking down the *psbU*, the recombinant *Synechocystis* cell has higher Chl *a* content, which can compensate for the decreased photosynthetic efficiency of the pigments. In summary, our work provides experimental evidence to complement the previously-reported understandings of the physiological roles of PsbU, which, more importantly, highlights the importance of monitoring the cyanobacterial biomass from different aspects, particularly the cell numbers, to better interpret the observed physiological behaviors of the cyanobacteria.

## Experimental procedures

### Bacterial strains and growth conditions

The CRISPRi tools (23) were kindly shared by Prof. Paul Hudson (KTH, Sweden), where the dCas9 protein and sgRNA scaffold are also available as Addgene plasmids #73223 and #73224. Three recombined mutants were constructed *via* Golden Gate Cloning (30): *Syn_*dCas9, *Synechocystis* carrying the dCas9 protein as the control; *Syn_*dCas9_t, mutant targeting on the antisense strand of *psbU*; *Syn_*dCas9_nt, mutant targeting on the sense strand of *psbU*. Two integrative plasmids pGGC171 and pGGC172, carrying a kanamycin resistance cassette, were used. The sgRNAs, under the anhydrotetracycline (aTc) inducible promoter P_L22_, were integrated into the *slr2030 to 2031* neutral site. N20 sequences were chosen based on Doench *et al.* (31). Plasmids, primers and strain lists are provided in Tables S1–S3.

All strains were grown in YBG-11 medium (32) with 50 μg/ml spectinomycin at 30 °C, 50 μmol_photon_/m^2^/s, 150 rpm, 75% humidity and ambient CO_2_, where the dCas protein and sgRNAs were induced with 1 μg/ml aTc, 3 h after inoculation (initial OD_750_ of 0.05), unless stated otherwise. For plasmid construction, *Escherichia coli* DH5α was used and grown in LB at 37 °C or on solid LB plates containing 1.5% (w/v) agar in an incubator, with 100 μg/ml ampicillin, 50 μg/ml kanamycin or 25 μg/ml chloramphenicol for Level 0, one and two constructs, respectively.

For RNA extraction and RT-qPCR, strains were grown in a multi-cultivator (MC 1000-OD, Photo Systems Instruments, Czech Republic). Per glass tube with 80 ml of YBG-11 medium was inoculated from exponentially growing cultures (OD_750_ < 2.5) to a final OD_750_ ∼0.5. Cells were induced with 1 μg/ml aTc at inoculation, under continuous white light of 50 μmol_photon_/m^2^/s and 30 °C, bubbled with air. Samples (20 ml) were taken at 0, 6 and 24 h after induction for RNA extraction.

### Biomass quantification

#### Optical density

The optical density measurements were carried out using a spectrophotometer at a wavelength of 750 nm. Samples were diluted to the linear range of 0.05 to 0.2 for all measurements with fresh YBG11 medium as the blank.

#### Cell number

The cell numbers and sizes were measured using a Coulter Counter (Multi-sizer, Beckman). Briefly, 200 μl cell suspensions were mixed with 9800 μl running buffer, and the calculation was done for the size range of 1 μm – 6 μm.

#### Chlorophyll a (Chl a)

Chl *a* extraction was performed according to a detailed protocol described elsewhere (33), and the Chla content was calculated as follows:

(1) $${Chl}_{a}\left[\frac{\mu g}{mL}\right]=12.9447*\left(A_{665}-A_{720}\right)$$

### RNA extraction and RT-qPCR

For RNA extraction, 20 ml of cultures (OD_750_ = 0.5–0.9) were centrifuged at 4000*g* for 10 min. Afterwards, the pellets were collected and resuspended in 1 ml PGTX solution and then shock-frozen in liquid nitrogen (34). Samples were incubated at 65 °C for 10 min (mixing every 2 min), and chilled on ice for 5 min and then mixed with 700 μl chloroform. After 10 min at room temperature with intermittent mixing, samples were centrifuged (21 °C, 22,000*g*, 15 min). The upper phase was transferred to fresh tubes with chloroform, re-centrifuged, and the resulting aqueous phase mixed with ice-cold isopropanol and stored at −20 °C overnight. RNA was pelleted (12,000*g*, 4 °C, 30 min), washed with 70% ethanol, centrifuged (5 min), air-dried, and dissolved in nuclease-free water. RNA integrity was checked *via* agarose gel electrophoresis.

For qRT-PCR, 1 μg RNA was treated with ezDNAse (Thermo Fisher Scientific) and then reverse-transcribed using SuperScript IV First-Strand Synthesis System (Thermo Fisher Scientific) and random hexamer primers. The cDNA was diluted 1:100 and used for qRT-PCR reaction with PowerUp SYBR Green Mastermix (Thermo Fisher Scientific) on a StepOnePlus Real-Time PCR System (Thermo Fisher Scientific). The manufacturer’s instructions were followed in these steps. Primer sequences are listed in Table S4. The housekeeping gene *rnpB* (35) was used for normalization following the ΔΔCt method (36). Afterwards, relative transcript levels of target genes in induced strains were normalized to those in non-induced control strains.

### Oxygen evolution rate determination

Biomass in the exponential growth phase was collected and resuspended in 5 ml fresh BG11 medium with 50 mM Na_2_HCO_3_ in a 10 ml gas chromatography (GC) glass tube with an air-tight lid to reach a final OD_750_ of ∼0.5. The medium was gassed with N_2_ before experiments, and afterwards, the oxygen concentration in the headspace was measured using gas chromatography (TRACE 1310, ThermoFisher) with a TG-Bond MIsieve column (length: 30 m; inside diameter: 0.32 mm; film thickness: 0.3 mm; Thermo Fisher), following the settings described elsewhere (37). The oxygen contents in the gas and liquid phases were calculated using the Ideal Gas Law and Henry’s Law, respectively.

The total produced oxygen content was the sum of oxygen in gas and liquid phases, and was normalized to the time and biomass concentration to calculate the oxygen evolution rate. Detailed protocol and equations are provided in the supplementary material.

### Quantum yield measurement

The quantum yield Y(II) of PSII was measured using a pulse amplitude modulation fluorometer (MULTI-COLOR-PAM fluorometer) following the general procedure described by Grund *et al.* (38). The biomass samples were all diluted to a final OD_750_ of 0.2 before the measurement.

## Data availability

All data related to the contents of this paper are provided in the main manuscript or supplementary materials. The raw data can be provided upon request to the corresponding author.

## Supporting information

This article contains supporting information (39).

## Conflict of interest

The authors declare that they do not have any conflicts of interest with the content of this article.
