# Correction: The necessity of multi-parameter normalization in cyanobacterial research: A case study of the PsbU in Synechocystis sp. PCC 6803 using CRISPRi

**DOI:** 10.1016/j.jbc.2026.111453

**Published:** 2026-05-11

**Authors:** Maria Christine Veit, Ron Stauder, Yu Bai, Ragini Gabhrani, Matthias Schmidt, Stephan Klähn, Bin Lai

In Table 1, the unit for growth rate currently reads:

**Table 1:** Growth kinetics and morphological parameters of *Synechocystis* strains.

Strains	Growth rate μ [d^−1^]		Cell density per OD750 [10^6^ cells/ml]	Cell size [μm]	Chl *a* [pg/cell]
	OD750	Cell No.			
*Syn*_dCas9	1.66 ± 0.31	1.93 ± 0.26	48.73 ± 2.87	2.24	0.059 ± 0.005
*Syn*_psbU_t	1.71 ± 0.36	1.88 ± 0.18	48.38 ± 2.85	2.35	0.062 ± 0.005
*Syn*_psbU_nt	1.61 ± 0.10	1.67 ± 0.17	39.74 ± 2.55	2.49	0.080 ± 0.007

Growth rate μ [h-1]

It should read:

Growth rate μ [d-1]

The sixth sentence of the “Knocking down psbU increases cell size and Chl a content per cell” currently reads:

The maximum growth rate for the control strain was 1.94 ± 0.26 h-1 whereas strain Syn_psbU_nt showed a decreased growth rate of 1.67 ± 0.17 h-1 during exponential growth phase (phase I).

It should read:

The maximum growth rate for the control strain was 1.94 ± 0.26 d-1 whereas strain Syn_psbU_nt showed a decreased growth rate of 1.67 ± 0.17 d-1 during exponential growth phase (phase I).

