# Supplementary material for: The necessity of multi-parameter normalization in cyanobacterial research: A case study of the PsbU in Synechocystis sp. PCC 6803 using CRISPRi
Source: J Biol Chem. 2025 Sep 24;301(11):110763. doi: 10.1016/j.jbc.2025.110763 (PMC12590133; doi:10.1016/j.jbc.2025.110763)
Supplement: Supporting material [file mmc1.docx]

Supplementary materials

**The necessity of multi-parameter normalization in cyanobacterial research: A case study of the physiological role of PsbU in Synechocystis sp. PCC 6803 using CRISPRi**

Maria Christine Veit ^1^, Ron Stauder ^2^, Yu Bai (白羽) ^1^, Ragini Gabhrani ^1^, Matthias Schmidt ^3^, Stephan Klähn ^2^, Bin Lai (赖斌) ^1^*

^1^ BMBF Junior Research Group Biophotovoltaics, Department of Microbial Biotechnology, Helmholtz Centre for Environmental Research – UFZ, 04318 Leipzig, Germany

^2^ Molecular Biology of Cyanobacteria Group, Department of Solar Materials Biotechnology, Helmholtz Centre for Environmental Research – UFZ, Leipzig 04318, Germany

^3^ Centre for Chemical Microscopy (ProVIS), Department of Technical Biogeochemistry, Helmholtz Centre for Environmental Research – UFZ, Leipzig 04318, Germany

* corresponding author: Dr. Bin Lai ([bin.lai@ufz.de](mailto:bin.lai@ufz.de))

**Experimental procedure**

**Oxygen evolution rate determination**

For each measurement, a sample volume of 100 μL was injected into an SSL injector with a split of 4 at 100 °C using the TriPlusRSH automated injection. The total GC batch runtime was 2.4 min. The glass tubes were incubated under standard conditions between the measurements.

The oxygen content in the gas phase was calculated based on the Ideal Gas Law, using the equation (2) below:

$n_{gas} (\mu mol)=\frac{{\%}_{O_{2}}*V1*1000}{100*\frac{V2}{n}}$ (2)

where V_1_ is the headspace volume of 5 mL, and $\frac{V2}{N}=24.7\frac{mL}{mmol}$, which is the volume-mass coefficient for gases at 30 °C, 1 atm pressure.

The amount of oxygen (µmol) in the liquid phase was calculated based on Henry’s Law with the equation (3) below:

$n_{liquid} (\mu mol)=\frac{{\%}_{O_{2}}*V1*1000}{100*{Ha}_{30^{\circ}C}}$ (3)

where V1 is the headspace volume of 5 mL and Ha_30°C_ is the Henry’s constant for oxygen at 30 °C of 858.3 $\frac{atm*mL}{mmol}$.

**Sample preparation for Helium Ion Microscopy**

The biological cells are chemically fixed on a substrate for helium ion microscopy imaging, following the procedure briefly described as follows.

A total of about 10^8^ cells in a total volume of 1 mL (measured by coulter counter) were mixed with 1 mL of 0.1 M sodium cacodylate buffer (pH of 7.2-7.4) containing 5% glutaraldehyde, and the mixture was kept steadily at 4 °C overnight. Afterwards, the cell suspension was gently filtered, to avoid cell disruption, with a hand filtration device and a syringe onto a 0.22 µm polycarbonate filter (Whatman, 25 mm diameter) sputter-coated with a 30 nm gold:palladium (80:20) layer. The filter was then rinsed by 5 mL fresh sodium cacodylate buffer to remove residues. Subsequently, the samples were treated with 1% hydrogen peroxide in sodium cacodylate buffer for 30 min, and then proceeded for dehydration with sequential ethanol wash at different concentration. The gradients (v/v%) of ethanol solutions are 30%. 50%, 70%, 80%, 90% and 95%. The filter containing the cells was submerged in each ethanol solution for 2 min, before moving to the next concentration. Afterwards, the filter was subsequently kept in pure ethanol, 1:1 (v/v) ethanol:hexamethyldisilazane (HMDS), and pure HMDS, with each condition for 10 min. The sample was finally air-dried under the fume hood for overnight, and then ready for microscopy imaging.

**Helium ion microscopy**

Helium ion microscopy imaging of the samples was carried out with a Zeiss Orion NanoFab (Zeiss Microscopy, Peabody, MA, USA) scanning helium ion microscope ^[[1]](#footnote-1)^. For excitation a beam of singly charged helium ions with an ion landing energy of 25 keV was scanned across the sample. The beam current was about 0.3 pA. The dwell time of the ion beam on each pixel was set to 1 µs. For imaging secondary electrons were collected with an Everhart-Thornley detector. In order to obtain a better signal-to-noise-ratio line averaging (8, 16 or 32 times, depending on the field-of-view) employed. For ion-milling of the bacterial cells singly charged neon-ion ions were used instead of helium-ion because of the greater mass and in turn higher material removal rates. The Ne-ion landing energy also amounted to 25 keV, the beam current was about 0.6 pA. In this work it was found that under these conditions an ion dose of 4 nC/µm^2^ suffices to mill through a cell of *Synechocystis sp* PCC6803. Milling was carried out in flat geometry and the analysis of the so prepared surfaces was done with He-ions and the stage tilted by 45 degrees. For milling it is crucial to ensure that the sample-temperature under Ne-beam does not increase to the point of deterioration of the sample. The parameters for the application in this work with the above-mentioned beam current were found to be 3 nm pixel spacing and a dwell-time of 0.5 µs.


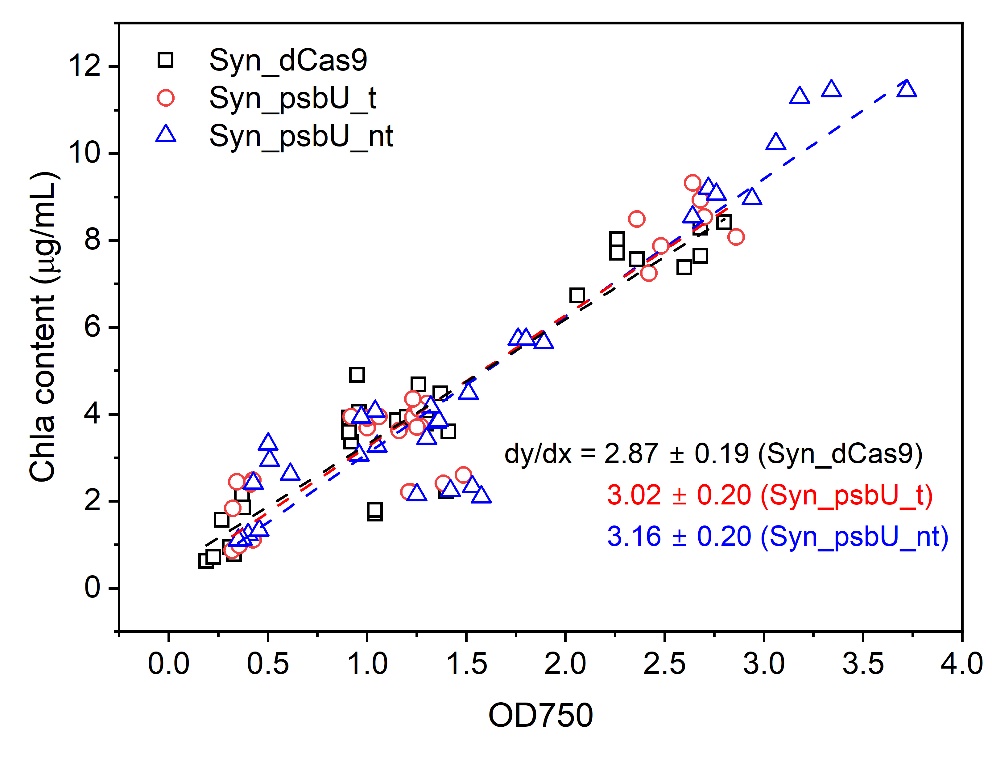


Figure S1 Correlation of Chl *a* content and optical density. Data were collected from 4 biological replicates for each strain.


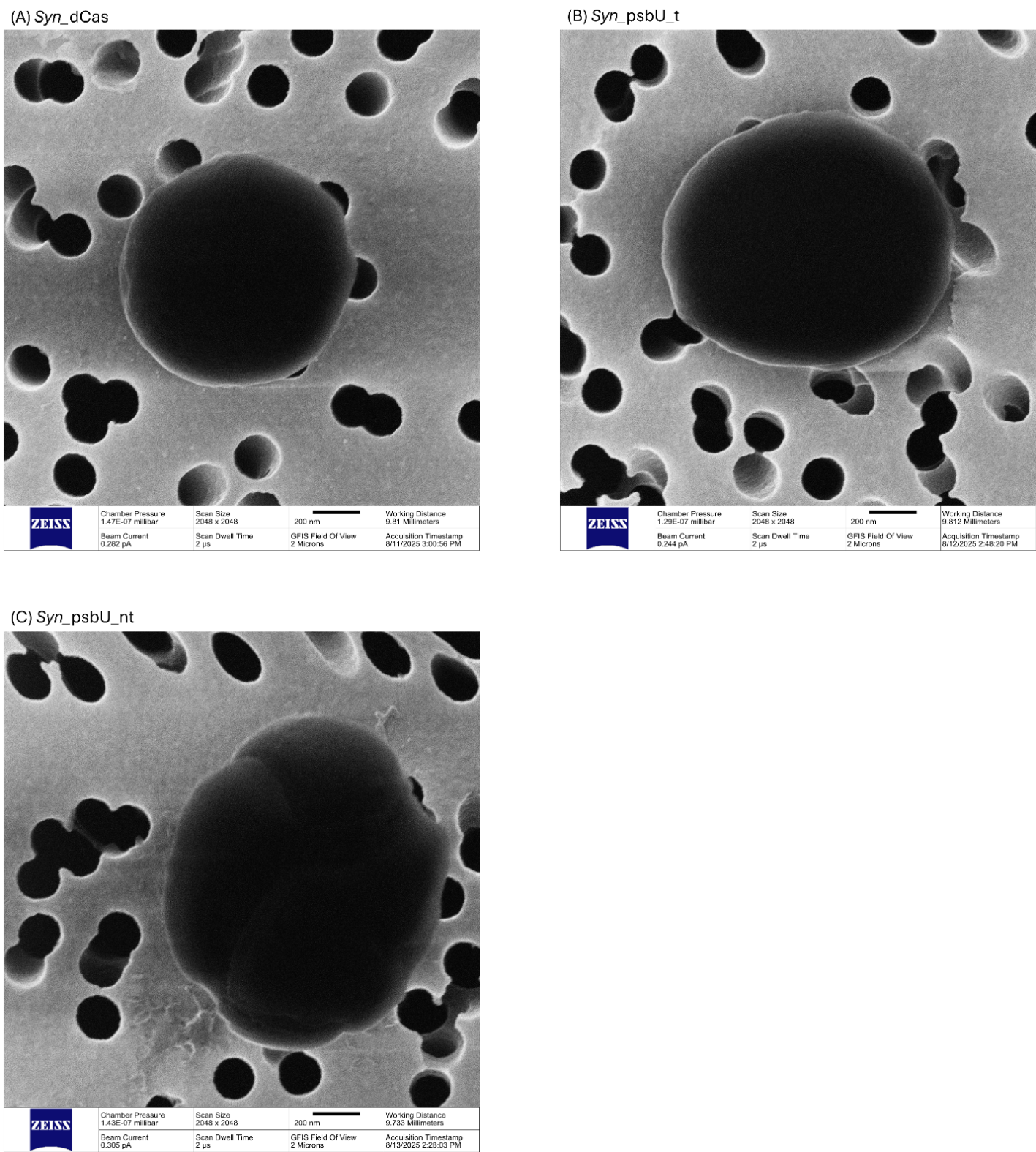


Figure S2 Helium ion microscopy of the *Synechocystis sp*. PCC6803 strains. Both knock-down strains showed relatively larger sizes compared to the dCas9 control strain. The knock-down mutant targeting on the non-template strand (*Syn*_psbU_nt) also exhibited more roughness surface.

Table S1. Primers used in this work and their purpose. Blue nucleotides indicate the position of the *Bsa*I restriction recognition site. Red nucleotides indicate the position of the N20 sequences.

| Primer | Sequence [5’-3’] | Purpose |
| --- | --- | --- |
| P_forw_1 | AAAGGTCTCGGATGGCAATAACCGTTGTCCCTTTTG | Amplification of the 5’ homologous region of the *slr 2030/2031* neutral site in *Synechocystis* and removal of an internal *Bpi*I restriction site |
| P_silent_mut_forw | AAAGGTCTCTGCCATTACGAGGACTATCCC |  |
| P_silent_mut_rev | AAAGGTCTCTTGGCGCAAAGGTTGACGGGCAATG |  |
| P_rev_1 | AAAGGTCTCGCTTTTTAGCAATGGCGGGAGACAG |  |
| P_forw_2 | AAAGGTCTCGGATGGTGGTCATTCTCAAGGAGTTGG | Amplification of the 3‘ homologous region of the *slr 2030/2031* neutral site in *Synechocystis* |
| P_rev_2 | AAAGGTCTCGCTTTAGCTCCTTCCGTATCCAGG |  |
| P_sgRNA_forw | AAAGGTCTCGGTTTTAGAGCTAGAAATAGCAAG | Amplification of the sgRNA scaffold and Kan^R^ cassette of Addgene plasmid #73224 |
| P_Kn_rev | AAAGGTCTCGCTTTCAACAAAGCCACGTTGTG |  |
| P_Tet_forw | AAAGGTCTCGGATGGAATTCGCGGCCGC | Amplification of the N20 sequences of the targeted genes in the PSII and the tet operator of Addgene plasmid #73224 |
| P_psbU_t_rev | AAAGGTCTCGAAACTGGTCAATTTAGCATCCACATAGCTCCCAGTATCTCTATC |  |
| P_psbU_t_rev | AAAGGTCTCGAAACACTGGTTGCGTGTAGCTTATTAGCTCCCAGTATCTCTATC |  |
| P_forw_cPCR | CCCCTTGCCCCATAAG | Colony PCR of genome-integrated sgRNAs with Kan^R^ cassette in *Synechocystis* *psbU* knockdown mutants |
| P_rev_cPCR | CACATAGACGCTGACTC |  |
| Seq_Level_0+1_for | AATGCAGCTGGCACGACAGG | Sequencing of Level 0 pGGC vectors |
| Seq_Level_0+1_rev | GGAGCAGACAAGCCCGTCAGG |  |
| P_Seq_KanR_rev | CCAGGTATTAGAAGAATATCCTC | Sequencing of Level 1 pGGC vectors |
| P_Seq_forw | TCAAGCTTTGCAAAAGGAATTTG | Sequencing of Level 2 pGGC vectors |
| P_Seq_forw_2 | TACAACCAATTAACCAATTCTG |  |
| P_Seq_rev | CCAATTCCCGATCCTGAC |  |

Table S2. Plasmids used for Golden Gate Cloning.

| **Plasmid** | **Properties for Golden Gate Cloning** | **Level** |
| --- | --- | --- |
| pGGC 0 | LacZ fragment flanked first by *Bsa*I, then *Bpi*I restriction sites on both ends | Level 0 |
| pGGC 3 | LacZ fragment flanked first by *Bpi*I, then *Bsa*I restriction sites on both ends | Level 1 Pos. 3 |
| pGGC 4 |  | Level 1 Pos. 4 |
| pGGC 5 |  | Level 1 Pos. 5 |
| pGGC 44 | *Bsa*I restriction sites on both ends of the end linker sequence | Level 1 EL 6 🡪7 |
| pGGC 139 | *Bsa*I restriction sites on both ends of the end linker sequence | Level 1 EL 1 🡪3 |
| pGGC 48 | LacZ fragment flanked by *Bsa*I restriction sites on both ends | Level 2 |

**Table S3. Bacterial strains used in this work, their purpose and properties. The bacterial strain *Syn_*dCas9 was a gift from Paul Hudson.** Further details are depicted in the plasmid charts provided as S2.

| Organism | Strain | Properties | Purpose | Reference |
| --- | --- | --- | --- | --- |
| *Escherichia coli* DH5α | pGGC 171 pGGC 172 | dlacZ Delta M15 Delta(lacZYA-argF) U169 recA1 endA1 hsdR17(rK-mK+) supE44 thi-1 gyrA96 relA1 | Assembly of CRISPRi constructs | This work |
| *Synechocystis* sp. PCC6803 | *Syn_*dCas9 | Suicide vector pMD19T-psba1-TetR-PL22-dCas9-SpR (Addgene plasmid #73223)  Sp^R^; Amp^R^; dCas9 under P_L22_ control | Genome integration of sgRNAs into the *slr 2030/2031* neutral site | (Yao et al. 2016) |
|  | *Syn*_psbU_t | Integrative vector pGGC 171  CRISPRi under P_L22_ control targeting the template strand of *psbU;* Kan^R^; Sp^R^ |  | This work |
|  | *Syn*_psbU_nt | Integrative vector pGGC 172  CRISPRi under P_L22_ control targeting the non-template strand of *psbU;* Kan^R^; Sp^R^ |  |  |

**Table S4 Primers used for RT-qPCR analysis.**

| **Primer** | **Sequence 5’ -> 3’** | **Primer target** |
| --- | --- | --- |
| # 297_F | GCCACAGAAAAATACCGCCC | *rnpB* houskeeping gene in *Syn*6803 genome |
| # 298_R | CACCTTTGCACCCTTACCCT |  |
| # 338_F | CTTGGAAGCCAATTTGGGTAG | 3’ region of *psbU* gene in *Syn*6803 genome |
| # 339_R | TAATACGGTCATCGCCAGAAG |  |
| # 342_F | CTAATAAGCTACACGCAACCA | gRNA region on plasmid pGGC172 |
| # 343_R | TTCAAGTTGATAACGGACTAGC |  |
| # 344_F | TGTGGATGCTAAATTGAC | gRNA region on plasmid pGGC171 |
| # 345_R | AACGGACTAGCCTTATTT |  |

1. Schmidt, M.; Byrne, J. M.; Maasilta, I. J. *Beilstein J. Nanotechnol.* **2021,** *12,* 1–23.  [↑](#footnote-ref-1)
